# CircNF1 promotes gastric cancer metastasis by stabilizing HMGA2 mRNA through IGF2BP1 interaction

**DOI:** 10.3389/fimmu.2026.1767319

**Published:** 2026-02-17

**Authors:** Yingying Sun, Yanli Ge, Zhiyu Xia, Cheng Guo, Junjie Zhang, Zhirong Wang, Zhe Wang

**Affiliations:** 1 Department of Gastroenterology, The Second Qilu Hospital of Shandong University, Jinan, China; 2 Department of Gastroenterology, Tongji Hospital, School of Medicine, Tongji University, Shanghai, China; 3 Endoscopy Center, Department of Gastroenterology, Shanghai East Hospital, Shanghai, China; 4 Department of Gastroenterology, Second Affiliated Hospital of Xinjiang Medical University, Urumqi, China

**Keywords:** circNF1, gastric cancer, HMGA2, IGF2BP1, metastasis, ZNF460

## Abstract

**Introduction:**

Gastric cancer (GC) is the fifth most common malignancy worldwide, with metastasis being the primary cause of mortality. Although circular RNAs (circRNAs) are implicated in GC pathogenesis, their specific roles in metastasis remain unclear. This study was designed to investigate the functional significance and underlying molecular mechanisms of circNF1 in GC metastasis.

**Methods:**

The expression of circNF1 was assessed in GC tissues and paired adjacent normal tissues using *in situ* hybridization, and its clinical relevance was evaluated via Cox regression analysis. Functional characterization was performed using transwell migration assays and in vivo metastatic mouse models to determine the effects of circNF1 overexpression or knockdown on GC cell motility and lung metastasis. Mechanistic investigations included molecular interaction studies to explore the association between circNF1 and IGF2BP1, as well as transcriptional regulation assays to identify the upstream regulator of circNF1 biogenesis.

**Results:**

Overexpression of circNF1 significantly enhanced GC cell migration in vitro and lung metastasis in vivo, whereas knockdown of circNF1 suppressed these metastatic phenotypes. Mechanistically, circNF1 was found to interact directly with IGF2BP1, thereby stabilizing HMGA2 mRNA and promoting its expression. Furthermore, ZNF460 was identified as a transcriptional activator of the NF1 host gene, which in turn upregulated circNF1 expression.

**Discussion:**

Our findings demonstrate that ZNF460-mediated upregulation of circNF1 drives GC metastasis by acting as a molecular scaffold to interact with IGF2BP1 and stabilize HMGA2 mRNA. This study not only elucidates a novel regulatory axis in GC metastasis but also identifies circNF1 as a promising prognostic biomarker and potential therapeutic target for the treatment of metastatic GC.

## Introduction

Gastric cancer (GC) is the fifth most commonly diagnosed tumor and the third leading cause of cancer-related deaths globally (1, 2). Due to the absence of specific early clinical manifestations and the limited efficacy of current screening strategies, the majority of GC cases are identified at advanced stages, along with extensive metastasis (3). Metastasis contributes to nearly 90% of cancer-associated mortality and represents the predominant factor contributing to GC mortality (4). Despite considerable progress in elucidating the general mechanisms underlying cancer metastasis, the molecular determinants specific to GC progression and their therapeutic targets remain insufficiently defined.

Emerging evidence highlights the role of circular RNAs (circRNAs) in various stages of carcinogenesis, including initiation, promotion, progression, and chemoresistance across different types of malignancies (5). CircRNAs, a subclass of non-coding RNAs (ncRNAs), are produced through the back-splicing of precursor transcripts and are characterized by the lack of 5’ caps and 3’ polyadenylated tails (6). Unlike linear RNAs, their covalently closed circular structure increases stability and evolutionary conservation, making them potential biomarkers for disease (7–9). CircRNAs show altered expression patterns in several cancers and contribute to a range of functional effects (10, 11). CircSEPT9 has been shown to act as an oncogene in the progression of triple-negative breast cancer (TNBC), suggesting its relevance as a prognostic and therapeutic target in TNBC patients (12). In bladder cancer (BC), circFAM13B reduces immune evasion and enhances sensitivity to immunotherapy by suppressing glycolysis and the acidic tumor microenvironment, indicating its value as a predictive biomarker in BC patients (10). Despite such findings, the mechanistic contributions of circRNAs to GC metastasis remain incompletely defined.

RNA-binding proteins (RBPs) are proteins that selectively recognize and bind RNA molecules and are involved in regulating gene expression (13, 14). Multiple studies support their involvement in cancer metastasis. For example, the RBP Hu antigen R (HuR) is overexpressed in breast cancer and promotes metastasis by binding to its downstream target FOXQ1 (15). Moreover, CPEB3 inhibits the metastasis of hepatocellular carcinoma cells by binding to MTDH mRNA and suppressing its translation (16). With ongoing advances in circRNAs research, increasing attention has been directed toward their interaction with RBPs. CircITGB6 improves the stability of PDPN mRNA, a gene that promotes epithelial-mesenchymal transition (EMT), via direct interaction with IGF2BP3, therefore enhancing metastatic behavior in colorectal and lung cancers (17). Although previous work identified circNF1 as a contributor to GC progression (11), the RBP-dependent mechanisms by which it modulates metastasis remain poorly understood and need further investigation.

This study demonstrates that circNF1 promotes GC metastasis *in vitro* and *in vivo*. The metastasis-promoting effect is mainly mediated by its direct interaction with insulin-like growth factor-2 mRNA-binding protein 1 (IGF2BP1), stabilizing high mobility group protein A2 (HMGA2) mRNA and enhancing metastatic activity. Further, zinc finger protein 460 (ZNF460) was found to promote the biogenesis of circNF1 by increasing NF1 transcription. These results provide insight into the mechanism by which circNF1 contributes to metastasis and offer potential directions for therapeutic intervention in GC.

## Materials and methods

### Patient samples and tissue collection

The GC tissues and adjacent non-tumorous tissues were collected from patients who underwent surgical resection at Shanghai Tongji Hospital. Immediately after resection, tissue specimens were snap-frozen in liquid nitrogen and stored until further analysis. Clinical and pathological data were obtained from the institutional medical records. All patients provided informed consent before participation in the study. The study was approved by the Medical Ethics Committee of Shanghai Tongji Hospital.

### Cell culture

Human GC cell lines were obtained from the Cell Bank of the Chinese Academy of Sciences. The HGC-27 cell line was cultured in Roswell Park Memorial Institute 1640 (RPMI-1640) medium (Servicebio), enriched with 10% fetal bovine serum (FBS, FuHeng Biology). The AGS cell line was maintained in Kaighn’s Modified Ham’s F-12K medium (Servicebio) with 10% FBS (FuHeng Biology). All cells were incubated in a humidified atmosphere at 37 °C with 5% CO_2_.

### Plasmid construction, RNA interference, and cell transfection

The circNF1 overexpression construct was generated by amplifying full-length human circNF1 via PCR and cloning it into the pLO5-ciR vector (Geneseed Biotech, Guangzhou, China), which contains essential elements required for circRNA formation. For functional inhibition of circNF1, a small interfering RNA targeting the back-splice junction (si-circNF1) was designed and synthesized, as previously described (11). Expression plasmids for IGF2BP1, HMGA2, and ZNF460 were obtained from Miaoling Biology (Wuhan, China). SiRNAs targeting IGF2BP1 (si-IGF2BP1#1 and si-IGF2BP1#2) and HMGA2 (si-HMGA2) were synthesized by Sangon Biotech (Shanghai, China). An empty vector (EV) and non-specific siRNA were used as negative controls (NC). All transfections were performed using Lipofectamine 3000 (Invitrogen), following the manufacturer’s protocol. The sequences of all siRNAs are provided in **Supplementary Table S1**.

### RNA isolation, quantitative real-time PCR, and sanger sequencing

Total RNA was extracted using TRIzol reagent (Invitrogen) and reverse transcribed using the PrimeScript™ RT Master Mix (Takara), as per the manufacturer’s instructions. Quantitative real-time PCR (qRT-PCR) was carried out using TB Green Premix Ex Taq™ II (Takara) on a QuantStudio 6 platform (Life Technologies). Sanger sequencing was used to confirm the back-splice junction of circNF1. All primers used in this study are listed in **Supplementary Table S1**.

### Western blot analysis

Total protein was isolated using Radio-Immunoprecipitation Assay (RIPA) buffer (NCM Biotech) enriched with protease inhibitor cocktail and phosphatase inhibitor cocktail (APExBIO). Protein concentrations were determined using the Bicinchoninic Acid (BCA) assay kit (Beyotime). Equal amounts of protein lysates were separated by Sodium Dodecyl Sulfate-Polyacrylamide Gel Electrophoresis (SDS-PAGE, Beyotime) and transferred onto Polyvinylidene Fluoride (PVDF) membranes (Immobilon). Membranes were blocked with 5% skim milk for 1 h at room temperature (RT), followed by overnight incubation at 4 °C with primary antibodies specific to target proteins. These membranes were washed thrice with Tris-Buffered Saline containing Tween 20 (TBST, Servicebio) and incubated for 1 h at RT with horseradish peroxidase (HRP)-conjugated goat anti-mouse or anti-rabbit IgG secondary antibodies (Beyotime). Immunoreactive bands were visualized using an Enhanced Chemiluminescence (ECL) detection system (Epizyme). Detailed antibody information is provided in **Supplementary Table S2**.

### Nuclear and cytoplasmic fractionation

Nuclear and cytoplasmic fractions were isolated using the Nuclear and Cytoplasmic Protein Extraction Kit (Beyotime) based on the manufacturer’s instructions. RNA was extracted from both fractions using TRIzol reagent (Invitrogen). GAPDH and U6 were used as cytoplasmic and nuclear markers, respectively.

### RNA fluorescence *in situ* hybridization

RNA localization was investigated using the Ribo™ Fluorescent *In Situ* Hybridization Kit (RiboBio), as per the manufacturer’s protocol. Cy3-labeled probes specific for circNF1, 18S rRNA, and U6 were used for hybridization. Cell nuclei were counterstained with 4’,6-diamidino-2-phenylindole (DAPI, Beyotime). Fluorescence signals were observed under a fluorescence microscope. Probe sequences are listed in **Supplementary Table S1**.

### Hematoxylin and eosin staining and *in situ* hybridization

Paraffin-embedded tissue sections were heated at 60 °C, then dewaxed and rehydrated. Nuclear staining was performed using hematoxylin (Solarbio), and cytoplasmic components were stained with an eosin solution (Solarbio). The stained sections were mounted with neutral resin (Biosharp) for preservation.

*In situ* hybridization targeting circNF1 was conducted according to the manufacturer’s protocol. Sections hybridized with the circNF1 probe were incubated with an anti-Digoxin-alkaline phosphatase (AP) antibody (Roche), followed by staining with 3,3’-diaminobenzidine (DAB, Vector). The ISH staining score was determined by multiplying the staining intensity by the percentage of positive cells. Scores<4 were categorized as low expression, whereas scores ≥ 4 indicated high expression. Probe sequences are detailed in **Supplementary Table S1**.

### Immunofluorescence assay

GC cells were fixed with 4% paraformaldehyde (PFA) (Beyotime) for 20 min at RT. After blocking with 10% goat serum for 1 h at RT, cells were incubated overnight at 4 °C with the primary antibody. After PBS washes, cells were incubated with the appropriate fluorescently labeled secondary antibodies for 1 h at 37 °C in the dark. Nuclear staining was performed using DAPI at RT. Fluorescence images were captured using an inverted fluorescence microscope. Antibodies used in this assay are listed in **Supplementary Table S2**.

### RNA pull-down assay

Biotin-labeled RNA probes were incubated with streptavidin-conjugated magnetic beads (Invitrogen). The bead–probe complexes were incubated with AGS cell lysates. After five washes, the retained proteins were eluted and visualized by silver staining.

### RNA-binding protein immunoprecipitation

The RIP assay was performed using the PureBinding RNA Immunoprecipitation Kit (Geneseed Biotech, Guangzhou, China), as per the manufacturer’s guidelines. Magnetic beads were pre-incubated with anti-IGF2BP1 antibodies (Abcam) or IgG control antibodies (Proteintech). AGS cells were lysed in RIP lysis buffer, and the lysates were incubated with antibody-conjugated beads. The co-precipitated RNA was isolated using TRIzol reagent (Invitrogen) and analyzed via qRT-PCR.

### Wound-healing assay

Cells were seeded into 6-well plates and cultured until reaching full confluency. Uniform vertical wounds were induced using 10µL pipette tips, followed by washing with PBS to remove detached cells. The scratch areas were observed at 0, 24, and 48 h under microscope. Cell migration was quantified using ImageJ software.

### Transwell assay

Approximately 3 × 10^4^ cells suspended in serum-free medium were seeded into the upper chambers of 24-well transwell inserts (8.0 μm pore size, JET Biofil). The lower chambers were filled with RPMI-1640 medium containing 10% FBS to serve as a chemoattractant. After 24 h of incubation, cells that migrated to the lower membrane surface were fixed with 4% PFA and stained using 1% Crystal Violet (Beyotime). All migrated cells were quantified using ImageJ software.

### Actinomycin D treatment

HGC-27 and AGS cells were treated with 5 μg/mL ActD (MCE, China) for 0, 3, 6, 9, and 12 h. Total RNA was isolated at each time point, and relative mRNA expression levels were normalized to baseline (0 h). The mRNA half-life was calculated using GraphPad Prism software.

### Dual-luciferase reporter assay

A luciferase reporter vector containing either the wild-type (WT) or mutant (Mut) NF1 promoter was constructed by cloning the respective sequences into pGL3-basic vectors (Promega). These vectors were co-transfected with a ZNF460 overexpression construct (Miaoling Biology, Wuhan, China). After 36 h, firefly and Renilla luciferase activities were measured using a Dual-Luciferase Reporter Assay Kit (Promega), as per the manufacturer’s instructions.

### Animal experiments

Four-week-old female BALB/c nude mice were obtained from Vital River Laboratory (Beijing, China) and randomly divided into experimental groups. Each mouse received a tail vein injection of 1 × 10^6^ HGC-27 cells suspended in 100 μL of PBS. After six weeks, the mice were euthanized by CO_2_ asphyxiation at a flow rate of 20% of the chamber volume per minute, followed by cervical dislocation to ensure death. Subsequently, lung tissues were harvested and fixed in 4% PFA. Lung metastases were evaluated, and the tissues were paraffin-embedded and examined with H&E staining.

### Molecular docking

Molecular docking was performed using the HDOCK SERVER (http://hdock.phys.hust.edu.cn/) to analyze the interaction between RNAs and their target proteins. Docking results were visualized and interpreted using PyMOL software. Protein structures were retrieved from the Protein Data Bank (PDB, https://www.rcsb.org), while RNA structures were predicted using the 3DRNA web tool (http://biophy.hust.edu.cn/new/3dRNA/create).

### Statistical analysis

Statistical analyses were conducted using GraphPad Prism and SPSS software. Student’s t-test, one-way ANOVA, and Chi-square test were applied to compare differences among groups. Univariate and multivariate Cox regression models were used to assess the impact of clinical parameters on GC patient survival. Kaplan–Meier analysis was used for survival curves. *P* < 0.05 was considered statistically significant.

## Results

### Upregulation of circNF1 in GC and its association with poor prognosis

**Figure 1:**
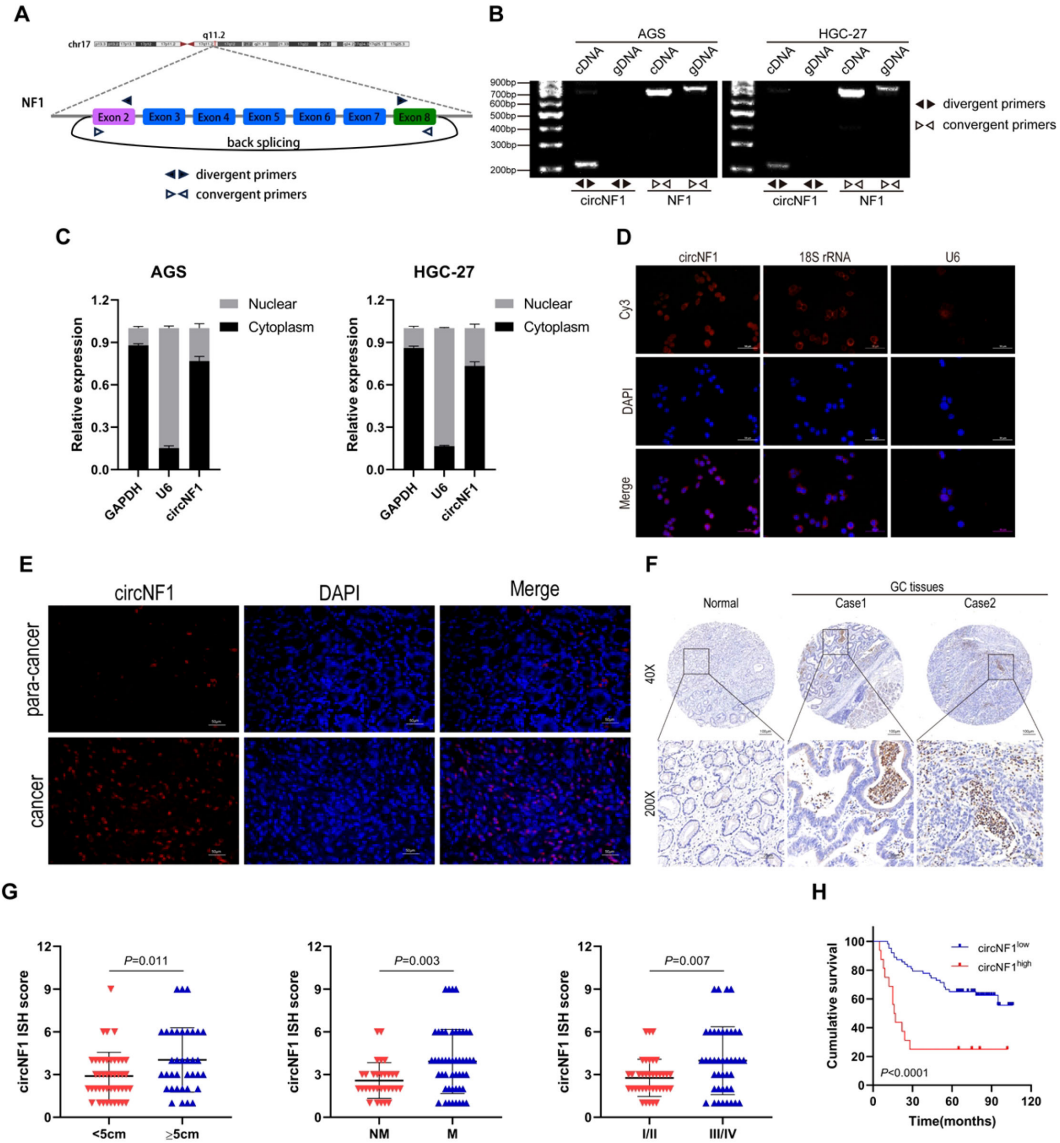
Upregulation of circNF1 in GC and its association with poor prognosis. **(A)** Genomic loci of circNF1. **(B)** Divergent primers detected circNF1 in cDNA but not in gDNA. **(C)** Subcellular localization of circNF1 assessed by qPCR assays. GAPDH (cytoplasm) and U6 (nucleus) transcripts were control markers. **(D)** CircNF1 localization was detected in AGS cells using FISH. 18 S rRNA and U6 were used as the cytoplasmic and nuclear markers, respectively. **(E)** CircNF1 expression was detected in GC tissues by FISH. **(F)** Representative images illustrating the expression of circNF1 in GC tissues as detected by ISH. **(G)** Dot distribution graph of circNF1 ISH staining scores was shown in 80 GC patients of different pathological characteristics. **(H)** Kaplan–Meier survival analysis showing that high circNF1 expression correlates with reduced cumulative survival in GC patients.

Our previous findings identified circNF1 as a circular RNA derived from exons 2 to 8 of the NF1 gene, comprising 828 nucleotides (**Figure 1A**) (11). In the present study, circNF1 amplification was observed exclusively in cDNA using divergent primers, whereas linear NF1 transcripts were amplified in both cDNA and gDNA using convergent primers, confirming its circular nature via back-splicing rather than genomic rearrangement or trans-splicing (**Figure 1B**). This result was further validated by RT-PCR and Sanger sequencing using divergent primers (**Supplementary Figure S1A**). Actinomycin D treatment revealed a longer half-life and higher stability of circNF1 compared to its linear counterpart (**Supplementary Figure S1B**). Nuclear-cytoplasmic fractionation demonstrated predominant cytoplasmic localization of circNF1 in AGS and HGC-27 cells (**Figure 1C**), a finding consistent with RNA FISH analysis (**Figure 1D**).

To determine the expression pattern and clinical relevance of circNF1, FISH and ISH assays were performed on GC tissues (**Figures 1E, F**), indicating significantly elevated circNF1 levels in tumor tissues relative to adjacent normal tissues. An analysis of 80 GC samples revealed a strong correlation between increased circNF1 expression and tumor size (*P* = 0.007), lymph node metastasis (*P* = 0.016), advanced clinical stage (*P* = 0.010), and poor survival (*P* = 0.010), with no significant association observed with age, gender, or tumor grade (**Table 1**). Moreover, ISH scores for circNF1 positively correlated with tumor size, lymph node involvement, and clinical stage (**Figure 1G**). Kaplan–Meier survival analysis demonstrated that patients with high circNF1 expression showed significantly shorter cumulative survival (**Figure 1H**). Multivariate analysis using the Cox proportional hazards model identified high circNF1 expression as an independent prognostic factor for poor outcome (HR = 2.281, *P* = 0.031; **Table 2**). These data collectively suggest that circNF1 was upregulated in GC and correlates with disease progression and unfavorable prognosis.

**Table 1:** Correlation between circNF1 expression and clinical pathological characteristics in 80 GC patients.

Characteristic	Number of cases	circNF1 expression		Chi-square	*P* value
		low	high		
Age, yr					
<60	33	27	6	0.116	0.733
≥60	47	37	10		
Gender					
Male	57	45	12	0.137	0.711
Female	23	19	4		
Grade					
G1/2	22	18	4	0.063	0.802
G3	58	46	12		
Tumor size					
<5 cm	44	40	4	7.273	0.007**
≥5cm	36	24	12		
Lymph node status					
No metastasis	31	29	2	5.806	0.016*
Metastasis	49	35	14		
Clinical Stage					
I/II	38	35	3	6.629	0.010*
III/IV	42	29	13		
Survival					
Alive	43	39	4	6.650	0.010*
Dead	37	25	12		

*P<0.05, **P<0.01.

GC, gastric cancer; yr, year; G, grade.

**Table 2:** Univariate and multivariate Cox regression analysis of circNF1 and survival in patients with GC.

Clinical variables	Univariate analysis		Multivariate analysis	
	HR (95% CI)	*P* value	HR (95% CI)	*P* value
Age, yr	1.083	0.811		
(<60 *vs*. ≥60)	(0.562-2.089)			
Gender	1.074	0.842		
(Male *vs*. Female)	(0.530-2.176)			
Grade	1.463	0.324		
(G1/2 *vs*. G3)	(0.687-3.113)			
Tumor size	3.523	<0.001***	2.256	0.058
(<5cm *vs*. ≥5cm)	(1.761-7.047)		(0.972-5.239)	
Lymph node status	6.606	<0.001***	4.388	0.056
(NM *vs*. M)	(2.544-17.149)		(0.961-20.036)	
Clinical Stage	5.234	<0.001***	1.175	0.816
(I/II *vs*. III/IV)	(2.370-11.556)		(0.300-4.602)	
circNF1 expression	3.577	<0.001***	2.281	0.031*
(low *vs*. high)	(1.777-7.201)		(1.080-4.820)	

*P<0.05, ***P<0.001.

GC, gastric cancer; yr, year; HR, hazard ratios; CI, confidence intervals.

### CircNF1 promotes GC cell migration and elicits tumor metastasis

**Figure 2:**
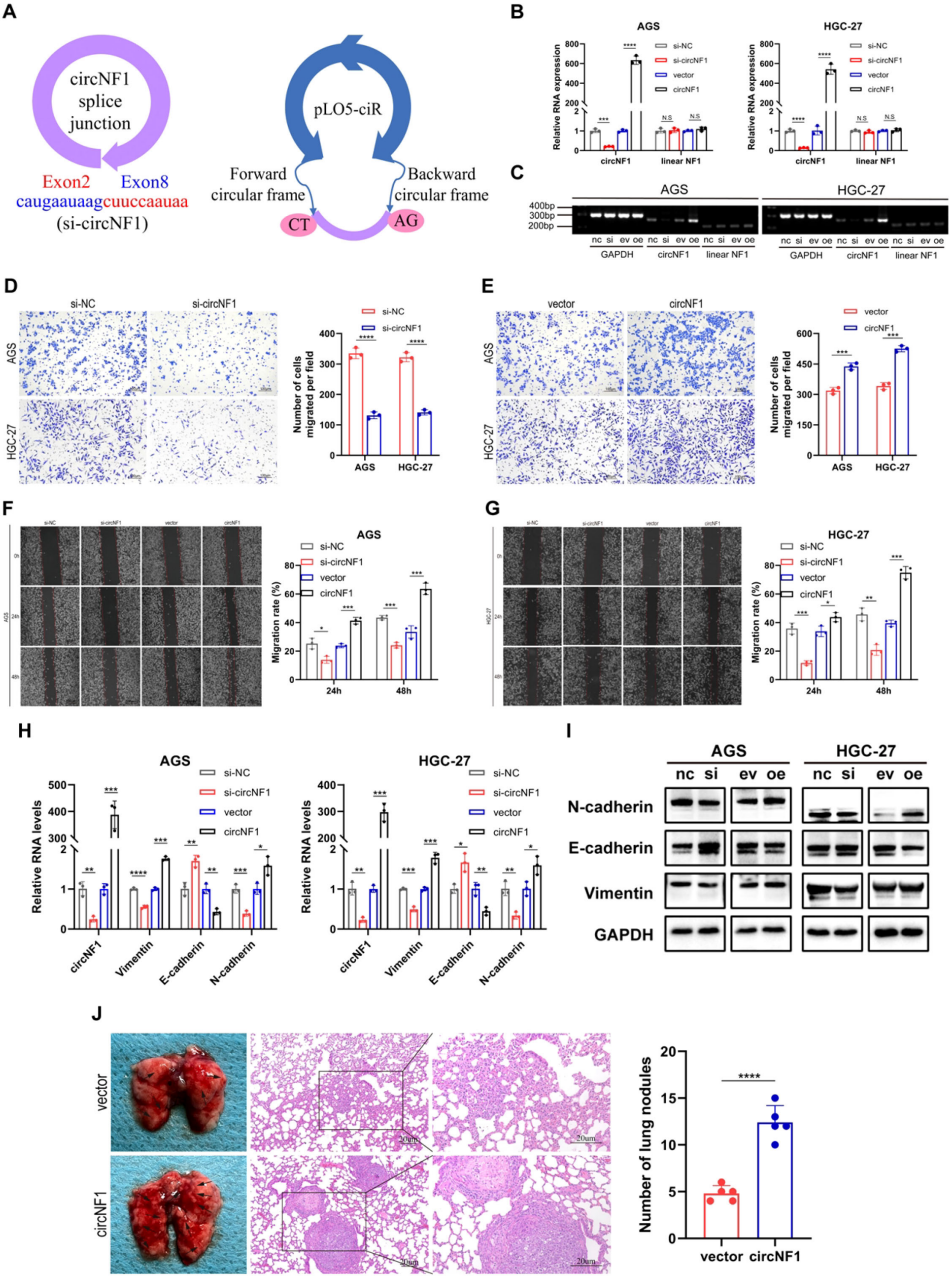
CircNF1 promotes GC cell migration *in vitro* and lung metastasis *in vivo*. **(A)** Schematic diagram of the si-circNF1 and circNF1 overexpression constructs. **(B, C)** qRT-PCR and RT-PCR analysis of circNF1 and linear NF1 expression in GC cells after transfection. **(D, E)** Transwell migration assay was used to assess the migratory capabilities of GC cells. **(F, G)** Wound healing assays evaluating GC cell migration. **(H)** Effects of circNF1 knockdown and overexpression on the RNA levels of relative markers. **(I)** Effects of circNF1 knockdown and overexpression on the protein levels of relative markers. **(J)** H&E staining of lung tissues from mice injected with GC cells; metastatic nodules are indicated by black arrows. si-NC/nc, small interfering RNA negative control; vector/ev, empty vector control. Data are shown as mean ± SD, *P < 0.05, **P< 0.01, ***P < 0.001, ****P < 0.0001.

To elucidate the functional role of circNF1 in GC progression, siRNAs targeting the back-splice junction of circNF1 and a circNF1 overexpression construct were developed (**Figure 2A**). Quantitative RT-PCR confirmed significant downregulation or overexpression of circNF1 in GC cells after transfection, while linear NF1 transcript levels remained unchanged (**Figure 2B**). PCR assays yielded consistent results (**Figure 2C**). Functional assays revealed that circNF1 knockdown suppressed, whereas its overexpression enhanced, cell migration as assessed by transwell and wound healing assays (**Figures 2D–G**). Moreover, circNF1 silencing increased mRNA expression of the epithelial marker E-cadherin and decreased mesenchymal markers N-cadherin and Vimentin; inverse trends were observed upon circNF1 overexpression (**Figure 2H**). Western blotting confirmed these changes at the protein level (**Figure 2I**). These findings indicate that circNF1 enhances the migration of GC cells, likely through the regulation of EMT-associated markers.

To further evaluate the metastatic potential of circNF1 *in vivo*, a lung metastasis model was developed by tail vein injection of HGC-27 cells with or without circNF1 overexpression into BALB/c nude mice. After six weeks, both the number and size of pulmonary metastatic nodules were markedly increased in the circNF1-overexpressing group compared to controls (**Figure 2J**). These results demonstrate that circNF1 promotes GC cell metastasis *in vivo*.

### CircNF1 interacts with IGF2BP1 to promote tumor metastasis

**Figure 3:**
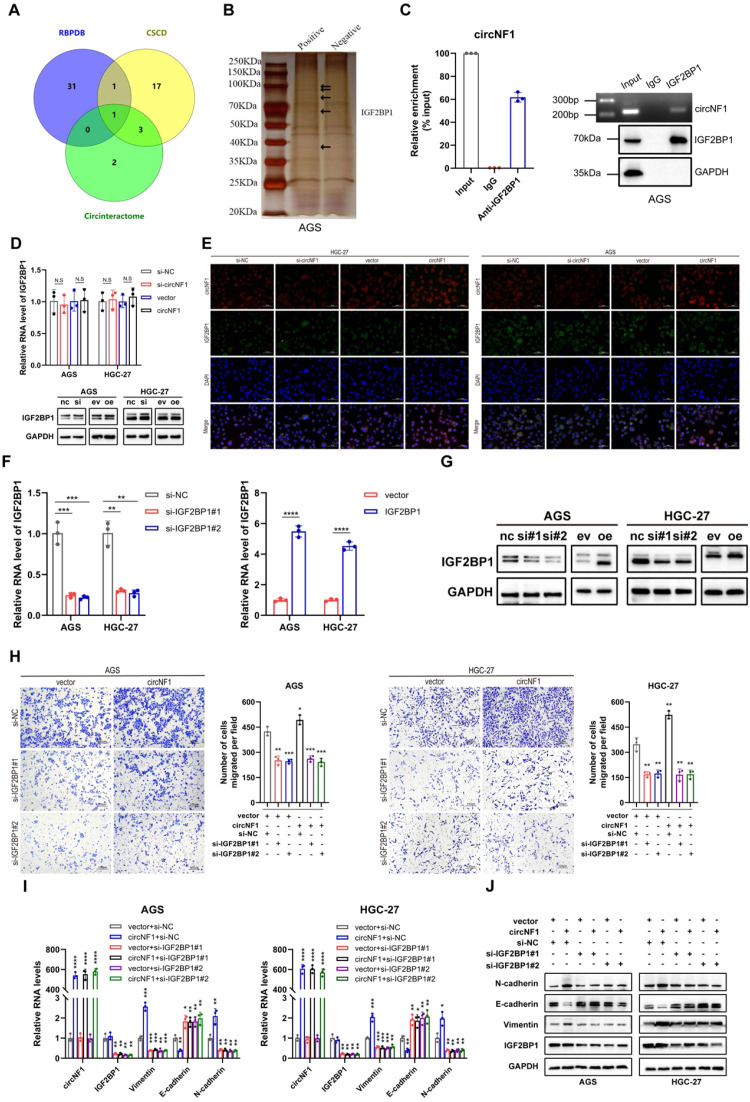
CircNF1 interacts with IGF2BP1 to promote tumor metastasis. **(A)** Venn diagram depicting potential RBPs. **(B)** Identification of interacting proteins via pull-down assays. Arrows indicate the additional band presented in the circNF1-protein complex. **(C)** RIP assays demonstrate the interaction of IGF2BP1 with circNF1. **(D)** The relative levels of IGF2BP1 mRNA and protein were quantified in GC cells after transfection with si-circNF1 and circNF1 overexpression plasmids. **(E)** IF-FISH assay demonstrates that circNF1 was colocalized with IGF2BP1 protein in the cytoplasm. **(F, G)** The relative mRNA and protein expression levels of IGF2BP1 were determined after transfection with si-IGF2BP1 and IGF2BP1 overexpression plasmids. **(H)** Transwell migration assay for circNF1 overexpressing GC cells with or without IGF2BP1 knockdown. **(I, J)** QRT-PCR and Western blot assays of relative markers. si-NC/nc, small interfering RNA negative control; vector/ev, empty vector control. Data are shown as mean ± SD, *P < 0.05, **P< 0.01, ***P < 0.001, ****P < 0.0001.

Considering the regulatory potential of circRNAs through RBP interactions, candidate RBPs for circNF1 were identified using Circinteractome (https://circinteractome.irp.nia.nih.gov), CSCD (http://gb.whu.edu.cn/CSCD), and RBPDB (http://rbpdb.ccbr.utoronto.ca) databases. IGF2BP1 was highlighted as a potential binding partner (**Figure 3A**). RNA pull-down assays showed enrichment of a ~70 kDa protein corresponding to IGF2BP1 (**Figure 3B**). RIP assays confirmed the direct interaction between circNF1 and IGF2BP1 (**Figure 3C**). However, qRT-PCR and Western blot analyses indicated no significant changes in IGF2BP1 expression after circNF1 knockdown or overexpression (**Figure 3D**). Co-localization of circNF1 and IGF2BP1 in the cytoplasm was validated through IF-FISH assays (**Figure 3E**). To assess the functional relevance of this interaction, siRNAs and an overexpression vector targeting IGF2BP1 were used (**Figures 3F, G**). The silencing of IGF2BP1 significantly reduced the circNF1-induced enhancement of cell migration (**Figure 3H**). In line with these observations, qRT-PCR and Western blotting revealed that the expression patterns of E-cadherin, N-cadherin, and Vimentin were also modulated after IGF2BP1 knockdown in circNF1-overexpressing cells (**Figures 3I, J**). These results establish IGF2BP1 as a key mediator of circNF1-driven motility in GC cells.

### CircNF1 stabilizes HMGA2 mRNA via its interaction with IGF2BP1

**Figure 4:**
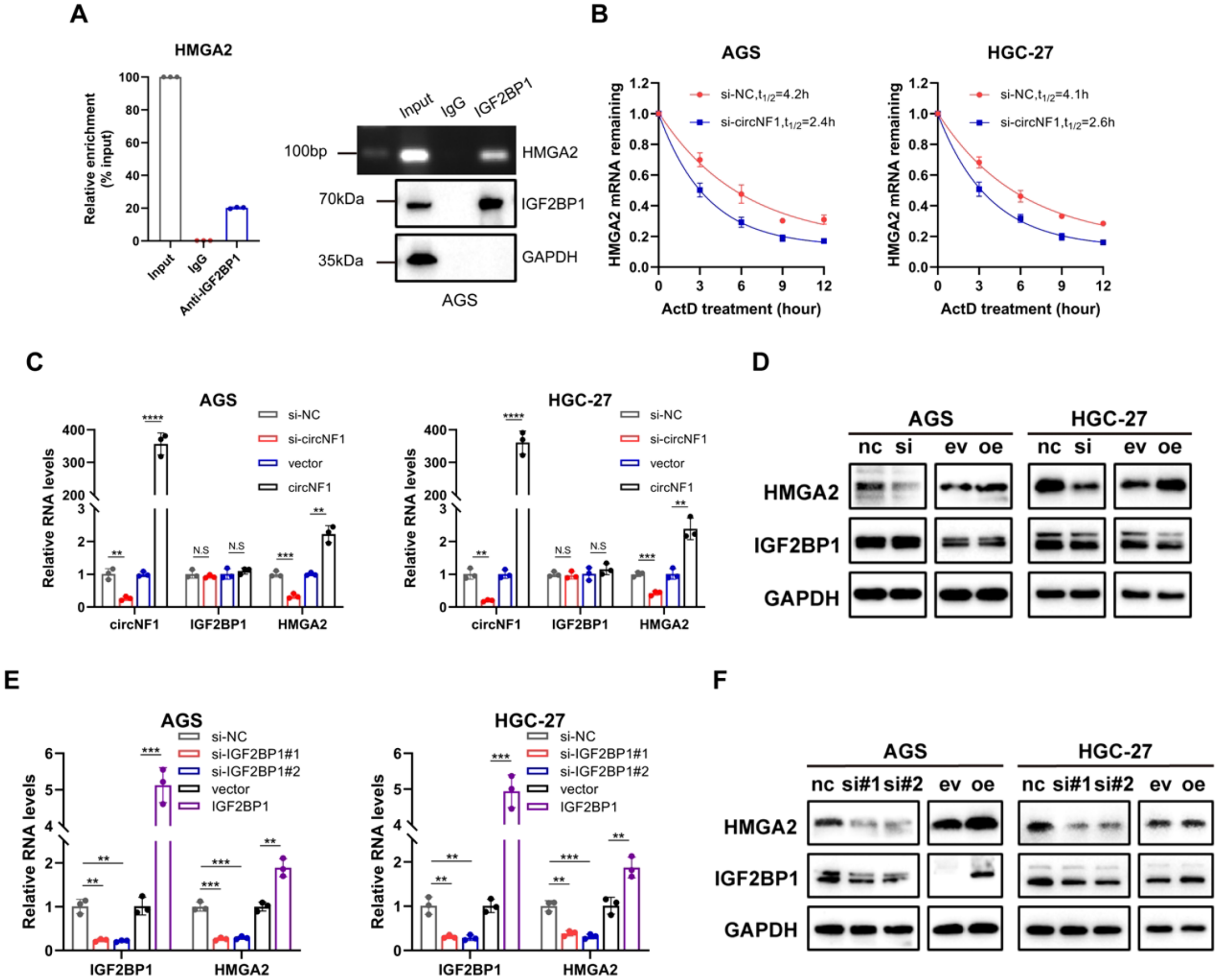
The crucial role of circNF1 and IGF2BP1 in maintaining HMGA2 mRNA stability. **(A)** RIP assay showing the connection between IGF2BP1 and HMGA2. **(B)** Measurement of the half-life of HMGA2 mRNA in GC cells treated with ActD for the indicated times. **(C, D)** Relative expression of HMGA2 was measured by qRT-PCR and Western blot assays in GC cells transfected with si-circNF1 and circNF1 overexpression plasmid. **(E, F)** Relative expression of HMGA2 was measured by qRT-PCR and Western blot assays in GC cells transfected with si-IGF2BP1 and IGF2BP1 overexpression plasmid. si-NC/nc, small interfering RNA negative control; vector/ev, empty vector control. Data are shown as mean ± SD, **P < 0.01, ***P < 0.001, ****P < 0.0001.

IGF2BP1 binds to multiple target mRNAs, including c-MYC, CD44, HMGA2, PTEN, and ACTB (10, 18–20). Among these targets, HMGA2 has been well-documented to be specifically associated with metastatic processes (21–23). Notably, bioinformatic analyses first predicted a high binding propensity between IGF2BP1 and HMGA2 (**Supplementary Figure S2**). RIP assays confirmed the binding of IGF2BP1 to HMGA2 mRNA (**Figure 4A**). CircNF1 knockdown resulted in decreased stability of HMGA2 mRNA (**Figure 4B**). However, circNF1 overexpression significantly elevated both mRNA and protein levels of HMGA2, while its silencing led to substantial downregulation (**Figures 4C, D**), consistent with results from Actinomycin D-based stability assays. To further confirm that HMGA2 is a downstream target of IGF2BP1, GC cell lines with IGF2BP1 overexpression or knockdown were generated. A positive correlation was observed between IGF2BP1 and HMGA2 expression; specifically, IGF2BP1 silencing reduced HMGA2 levels, whereas its overexpression enhanced them at both RNA and protein levels (**Figures 4E, F**).

**Figure 5:**
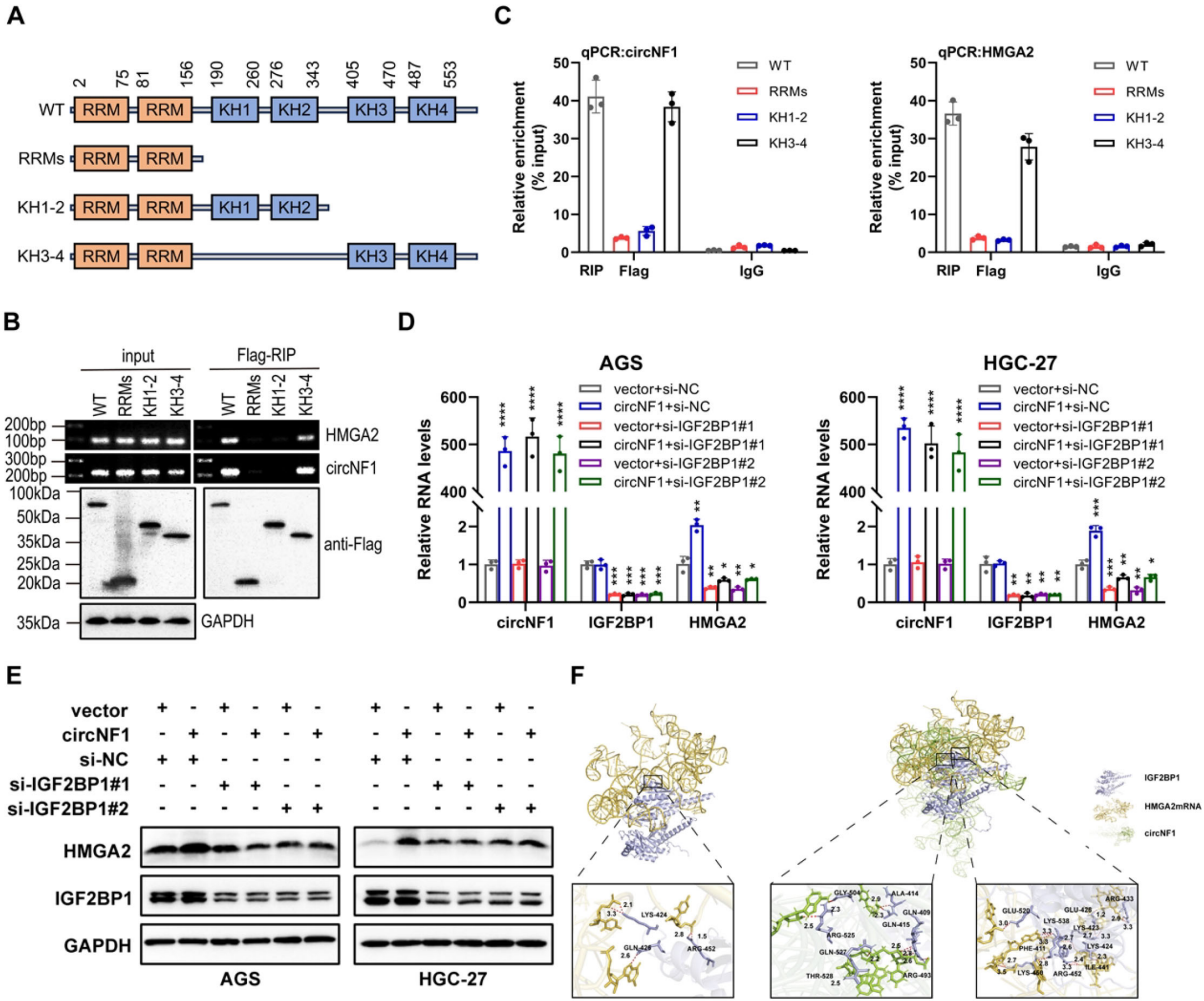
CircNF1 stabilizes HMGA2 mRNA via IGF2BP1 interaction. **(A)** Schematic structure showing RNA-binding domains within the IGF2BP1 protein and the list of different IGF2BP1 truncation mutants. **(B)** RT-PCR assays for the enrichment of circNF1 or HMGA2 mRNA in AGS cells expressing flag-tagged full-length or truncated mutants of IGF2BP1. **(C)** Relative enrichment representing the enrichment of circNF1 and HMGA2 associated with truncated IGF2BP1 compared to an input control. **(D, E)** QRT-PCR and Western blot assays of HMGA2 for circNF1 overexpression in GC cells with or without IGF2BP1 knockdown. **(F)** Molecular docking analysis predicting the binding sites of IGF2BP1 with circNF1 and HMGA2 mRNA. Data are shown as mean ± SD, *P < 0.05, **P < 0.01, ***P < 0.001, ****P < 0.0001.

Based on these findings, it was hypothesized that circNF1 modulates HMGA2 expression via its interaction with IGF2BP1. To investigate the interaction between IGF2BP1 and circNF1 as well as HMGA2 mRNA, FLAG-tagged truncation mutants of IGF2BP1 were constructed (**Figure 5A**). RIP assays showed that circNF1 and HMGA2 mRNA bind to the KH3–4 di-domain of IGF2BP1 (**Figures 5B, C**). Moreover, IGF2BP1 knockdown in circNF1-overexpressing cells decreased HMGA2 expression at both transcript and protein levels (**Figures 5D, E**). Molecular docking analysis further revealed that circNF1 enhances the interaction between IGF2BP1 and HMGA2 mRNA by increasing binding affinity (**Figure 5F**).

### CircNF1 promotes tumor metastasis via the HMGA2 pathway

**Figure 6:**
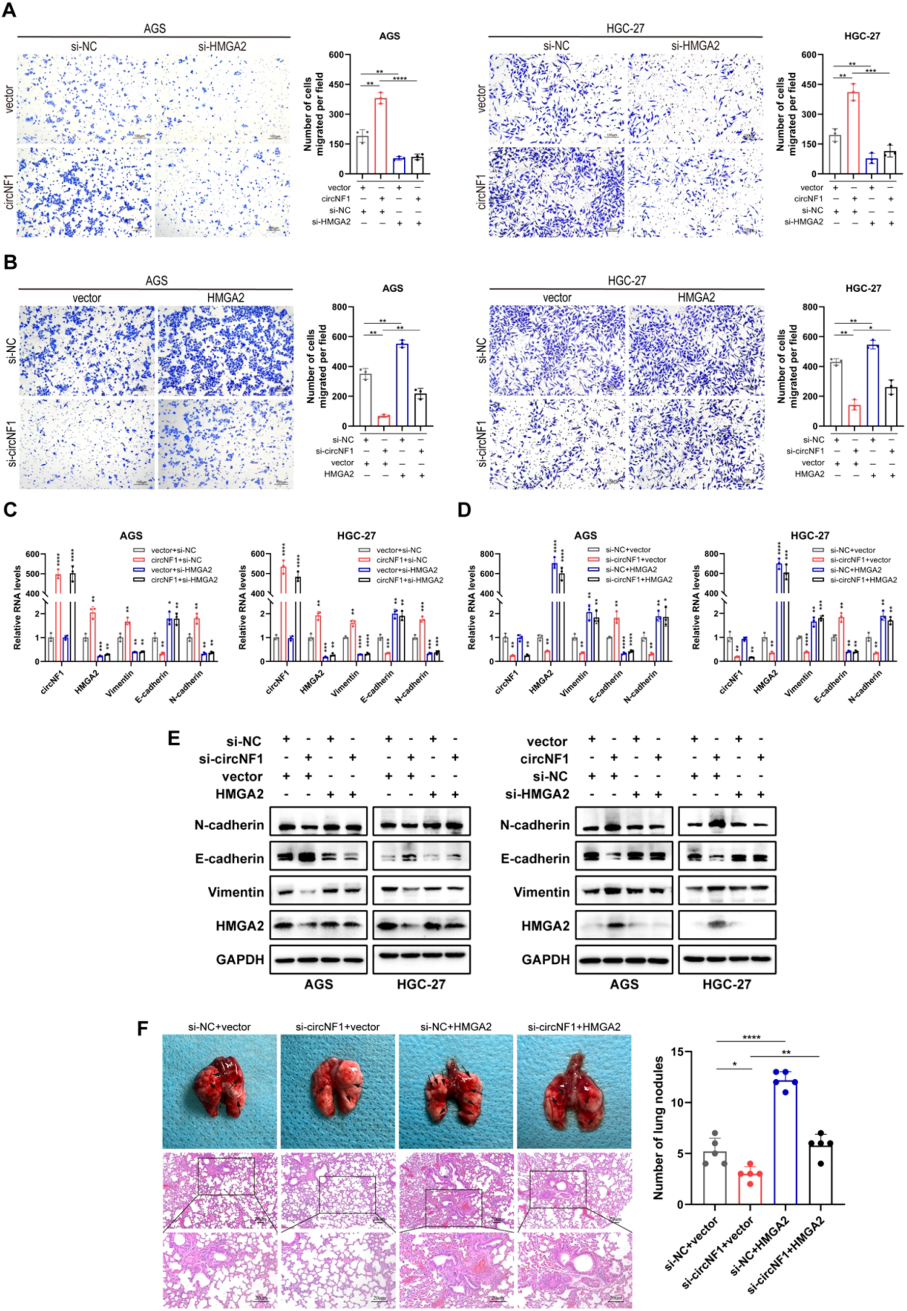
CircNF1 promotes tumor metastasis via the HMGA2 pathway. **(A, B)** Transwell migration assays assessing the migratory potential of GC cells co-transfected with the indicated vectors or siRNAs. **(C-E)** Expression of metastasis-related markers analyzed by qRT-PCR and western blot after co-transfection. **(F)** HMGA2 overexpression reversed the reduced lung metastasis induced by circNF1 knockdown in HGC-27 cells, as observed in tail vein-injected mice. Black arrows indicate metastatic nodules. Data are shown as mean ± SD, *P < 0.05, **P < 0.01, ***P < 0.001, ****P < 0.0001.

Rescue assays were conducted to evaluate whether the metastatic effects of circNF1 are mediated through the HMGA2 axis. *In vitro* transwell migration assays revealed that HMGA2 knockdown significantly attenuated the circNF1 overexpression-enhanced migration (**Figure 6A**). Conversely, HMGA2 overexpression rescued the suppressed migratory phenotype caused by circNF1 knockdown (**Figure 6B**). Moreover, HMGA2 silencing reversed the circNF1-induced EMT-like changes, as evidenced by altered expression of E-cadherin, Vimentin, and N-cadherin at both mRNA and protein levels (**Figures 6C–E**). *In vivo* analysis using a lung metastasis model further supported these findings. HMGA2 overexpression effectively reversed the reduction in metastatic nodules observed after circNF1 knockdown (**Figure 6F**). These data demonstrate that circNF1 exerts its pro-metastatic effects in GC via the HMGA2 signaling pathway.

### ZNF460 enhances NF1 and circNF1 expression

**Figure 7:**
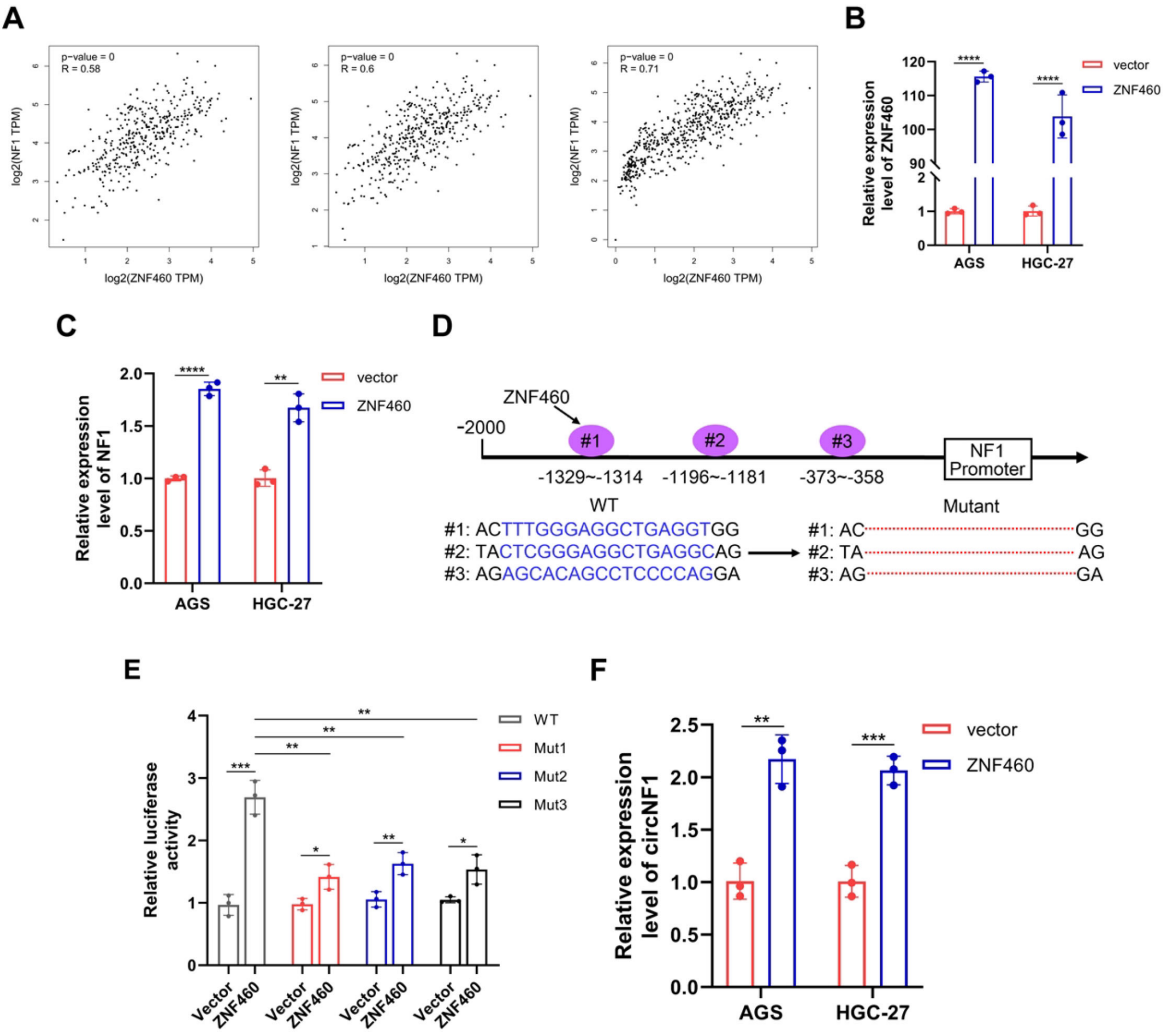
ZNF460 promotes the expression of NF1 and circNF1. **(A)** Positive correlation between ZNF460 and NF1 expression in GC tissues and adjacent non-cancerous tissues across datasets: TCGA STAD Tumor (left), TCGA STAD Tumor and Normal (middle), and TCGA STAD combined with GTEx Stomach (right). **(B)** The expression of ZNF460 was detected in GC cells transfected with ZNF460 overexpression plasmids by qRT-PCR. **(C)** Expression of NF1 was detected in GC cells transfected with ZNF460 overexpression plasmids by qRT-PCR. **(D)** Schematic illustration of wild type (WT) and mutant (Mut) sequences of the three putative binding sites of ZNF460 on NF1 promoter. **(E)** Luciferase reporter assay showing transcriptional activity of WT and Mut NF1 promoter constructs co-transfected with ZNF460 overexpression plasmids. **(F)** CircNF1 expression assessed by qRT-PCR in GC cells after ZNF460 overexpression. Data are shown as mean ± SD, **P < 0.01, ***P < 0.001, ****P < 0.0001.

To identify transcriptional regulators of NF1 and circNF1, a bioinformatic analysis was performed using the JASPAR database (https://jaspar.genereg.net/), which predicted several transcription factors potentially binding to the NF1 promoter. Among these, ZNF460 showed the highest prediction score, suggesting a prominent role in NF1 regulation. Correlation analysis using the GEPIA database (http://gepia.cancer-pku.cn) revealed a positive association between ZNF460 and NF1 expression levels in both GC and adjacent non-tumorous tissues (**Figure 7A**), supporting the initial prediction.

To further elucidate the role of ZNF460, a ZNF460 overexpression plasmid was constructed and its expression was validated via qRT-PCR (**Figure 7B**). Overexpression of ZNF460 in GC cells significantly upregulated NF1 mRNA expression (**Figure 7C**). Based on JASPAR predictions (https://jaspar.genereg.net/), six potential ZNF460 binding motifs were identified within the NF1 promoter region, from which three high-probability sites were selected for further functional analysis. Respective WT and Mut NF1 promoter constructs were generated and cloned into luciferase reporter vectors (**Figure 7D**). Dual-luciferase assays depicted that ZNF460 significantly increased the transcription of the WT NF1 promoter, with its binding not restricted to a single site (**Figure 7E**). To investigate whether ZNF460-mediated activation of the NF1 promoter also affected circNF1 expression, ZNF460 was overexpressed in GC cells. Next, qRT-PCR analysis indicated a marked elevation of circNF1 levels in ZNF460-overexpressing cells relative to vector controls (**Figure 7F**). These findings suggest that ZNF460 transcriptionally activates the NF1 promoter, enhancing circNF1 expression.

## Discussion

CircRNAs have emerged as pivotal regulators within the ncRNA landscape due to their covalently closed loop structures (24–26). However, the mechanisms underlying their dysregulation in GC metastasis remain poorly defined. The present study demonstrated that circNF1 is significantly upregulated in GC tissues, predominantly localized in the cytoplasm, and functions as an independent prognostic indicator. Functional assays revealed that circNF1 overexpression markedly enhances GC cell migration *in vitro* and promotes lung metastasis *in vivo*, whereas it silencing substantially attenuates these effects. Mechanistically, circNF1 interacts with IGF2BP1 to stabilize HMGA2 mRNA, a key metastasis-associated transcript, thus promoting GC progression. Moreover, ZNF460 was identified as a potential regulator of circNF1 biogenesis. These findings collectively highlight circNF1 as a candidate prognostic biomarker and therapeutic target in GC.

The biological functions of circRNAs are closely associated with their subcellular localization. Nuclear-localized intron-containing circRNAs may participate in transcriptional and splicing regulation (27, 28), while exon-derived circRNAs are predominantly cytoplasmic (29). Based on this pattern, circNF1—generated by backsplicing of exons 2–8 of the NF1 gene—was enriched in the cytoplasm. Among the diverse functions of exonic circRNAs, miRNA sponging is the most extensively characterized. Our previous study identified circNF1 as an oncogenic circRNA in GC that promotes proliferation by sponging miR-16 (11). Further studies confirmed its miRNA sponge activity in breast cancer and glioblastoma (30, 31). Moreover, recent evidence revealed that circNF1 binds to ANXA1 and inhibits its USP7-mediated deubiquitination, thus stabilizing PD-L1 and promoting immune evasion in esophageal squamous cell carcinoma (32). These findings provide novel mechanistic insights into the role of circNF1 in GC pathogenesis.

Emerging data indicate that the functions of circRNAs are often mediated through interactions with RBPs (33, 34). In this study, bioinformatic prediction suggested a potential interaction between circNF1 and IGF2BP1, which was further validated through RNA pull-down and RIP assays. As a conserved RBP (35), IGF2BP1 regulates mRNA stability, translation, and localization, affecting multiple cellular processes (36, 37). Its overexpression correlates with poor prognosis in colorectal, lung, and breast cancers (38–40). In the present study, IGF2BP1 knockdown abrogated circNF1-mediated metastatic effects in GC. However, the precise mechanisms underlying this regulatory axis need further research.

Previous studies have established that circRNAs can interact with RBPs through multiple mechanisms, such as functioning as protein scaffolds (41, 42), molecular decoys (41), or spatial recruiters (43). This study elaborated that circNF1 acts as a scaffold for IGF2BP1, reinforcing its binding to HMGA2 mRNA and thus extending transcript half-life to enhance stability. These findings were further supported by IF-FISH assays, which demonstrated that neither circNF1 overexpression nor depletion significantly changed IGF2BP1 expression levels or subcellular localization. IGF2BP1 is a multidomain RBP comprising two N-terminal RNA recognition motifs (RRMs) and four C-terminal K homology (KH) domains, which collectively confer its RNA-binding activity (44). Structural and functional studies have demonstrated that the KH domains, particularly KH3-4, are essential for stabilizing IGF2BP1-RNA interactions (45, 46). The present study highlights the mechanistic role of the KH3–4 domain in facilitating IGF2BP1’s binding to circNF1 and HMGA2 mRNA. Molecular docking simulations revealed spatially distinct binding sites for these RNA molecules on IGF2BP1, indicating a cooperative binding mechanism. HMGA2 is a non-histone architectural transcription factor that modulates gene expression by binding to AT-rich regions within the minor groove of B-form DNA, inducing chromatin remodeling (47). Previous studies have shown that HMGA2 promotes EMT in ovarian surface epithelial cells, with approximately 16% of its downstream targets related to EMT pathways (21). In this study, functional validation through *in vitro* and *in vivo* rescue assays conclusively established HMGA2 as the key downstream mediator of circNF1-IGF2BP1-driven metastatic progression in GC. These findings implicate the circNF1/IGF2BP1/HMGA2 signaling cascade as a potential therapeutic target for metastatic GC.

Back-splicing is the primary mechanism for circRNAs biogenesis, which critically depends on pre-mRNA participation (8). However, the upstream transcriptional regulation of circRNA biogenesis remains poorly understood (48, 49). In this study, bioinformatic analyses combined with dual-luciferase reporter assays identified ZNF460 as a transcription factor that directly binds to the NF1 promoter and enhances its activity. ZNF460, a DNA-binding transcriptional regulator, has been implicated in the progression of multiple human cancers (50, 51). Quantitative RT-PCR analysis revealed that ZNF460 overexpression markedly increased circNF1 levels, suggesting a role in promoting NF1 transcription and circNF1 biogenesis. However, the regulatory mechanisms controlling circNF1 formation remain incompletely defined and need further exploration.

## Conclusion

**Figure 8:**
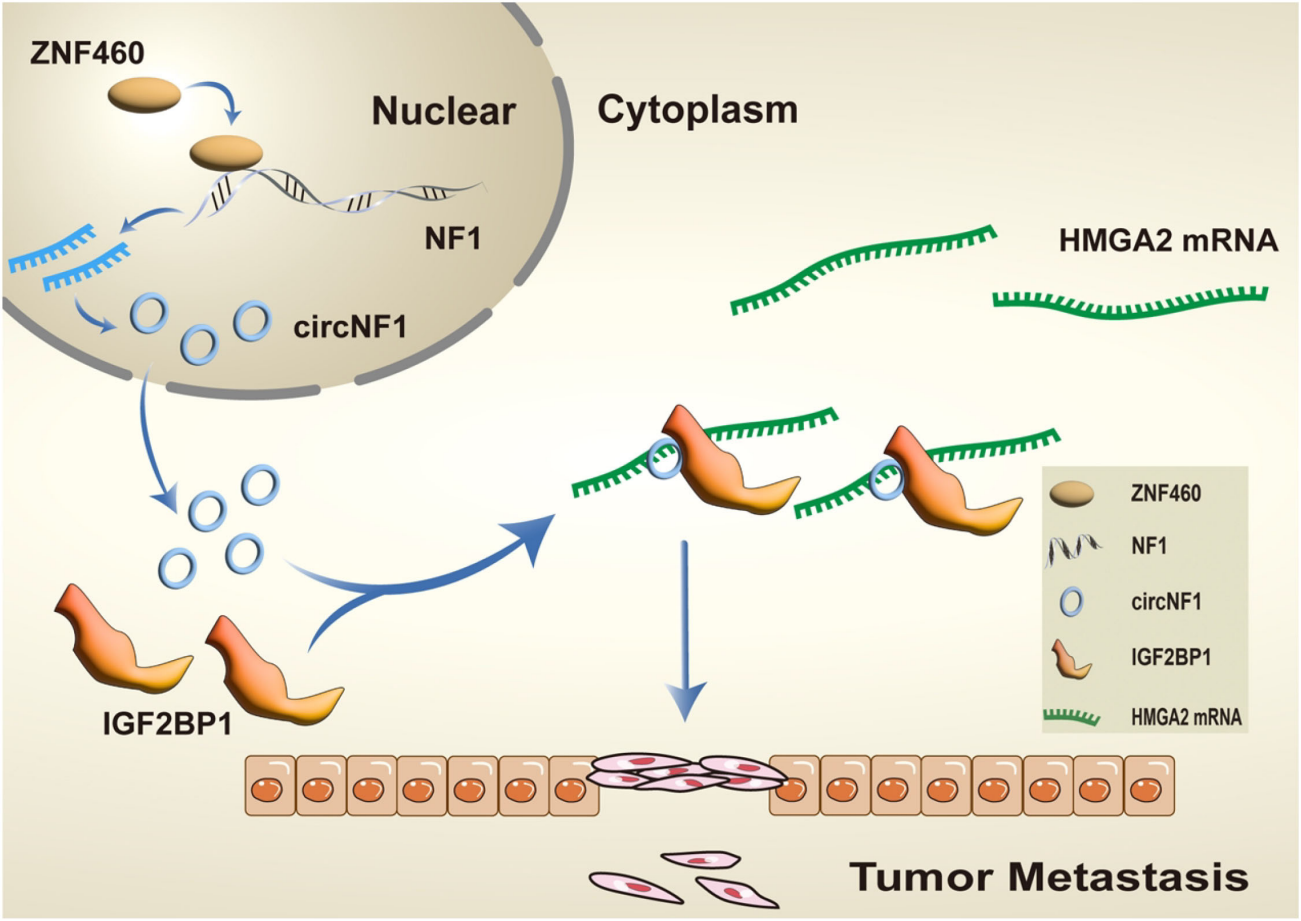
Schematic illustration of circNF1 molecular mechanism. CircNF1, regulated by ZNF460, promotes GC metastasis by binding to IGF2BP1 and stabilizing HMGA2 mRNA.

This study concluded that circNF1 is markedly upregulated in GC tissues and functions as an oncogenic regulator associated with adverse clinical outcomes. Functional analyses revealed that circNF1 enhances the migration and metastasis of GC cells *in vitro* and *in vivo*. Mechanistically (**Figure 8**), circNF1 promotes metastasis by interacting with the RNA-binding protein IGF2BP1, thus stabilizing HMGA2 mRNA. Furthermore, ZNF460 was identified as an upstream transcriptional regulator that promotes circNF1 biogenesis through activation of its host gene, NF1. These findings highlight circNF1 as a potential prognostic biomarker and therapeutic target in GC.

## Data Availability

The raw data supporting the conclusions of this article will be made available by the authors, without undue reservation.
