# Supplementary material for: CircNF1 promotes gastric cancer metastasis by stabilizing HMGA2 mRNA through IGF2BP1 interaction
Source: Front Immunol. 2026 Feb 17;17:1767319. doi: 10.3389/fimmu.2026.1767319 (PMC12953387; doi:10.3389/fimmu.2026.1767319)
Supplement: Supplementary file 1 [file DataSheet1.docx]

**Supplementary Figure 1**


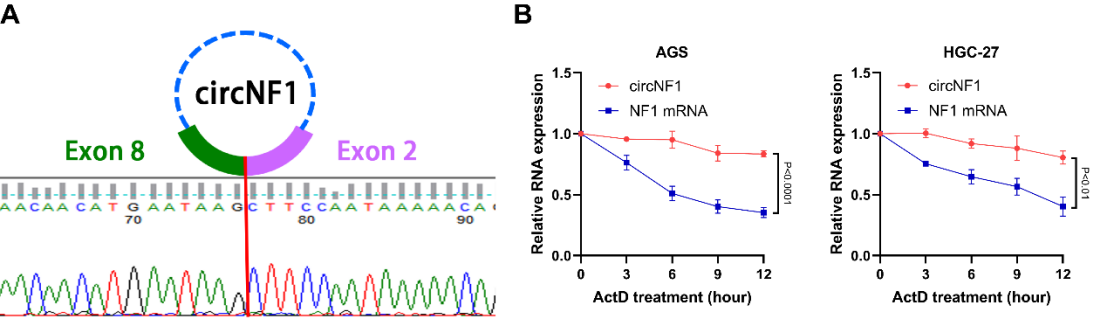
**Fig. S1 Characterization of circNF1. A** Sanger sequencing validation of back-splice junctions between exon 2 and exon 8 of NF1 gene. **B** QRT–PCR analysis for the expression of circNF1 and NF1 mRNAs after treatment with Actinomycin D.
