# Supplementary material for: CircNF1 promotes gastric cancer metastasis by stabilizing HMGA2 mRNA through IGF2BP1 interaction
Source: Front Immunol. 2026 Feb 17;17:1767319. doi: 10.3389/fimmu.2026.1767319 (PMC12953387; doi:10.3389/fimmu.2026.1767319)
Supplement: Supplementary file 2 [file DataSheet2.docx]

**Supplementary Table 1**

| **Table S1. Sequences of siRNAs, probes and primers** | |
| --- | --- |
| **Sequences of siRNAs, probes and primers** | |
| **siRNAs Used for Transduction** | |
| **siRNAs** | **Sequence (5’-3’)** |
| si-NC | UUCUCCGAACGUGUCACGUTT |
| si-circNF1 | CAUGAAUAAGCUUCCAAUAA |
| si-IGF2BP1#1 | ACGCUUAGAGAUUGAACAUUC |
| si-IGF2BP1#2 | GCUCCCUAUAGCUCCUUUATT |
| si-HMGA2 | AGUCCCUCUAAAGCAGCUCAA |
| **Probes Used for In Situ Hybridization** | |
| **Probes** | **Sequence** |
| circNF1 | TGTCCTGTTTTTATTGGAAGCTTATTCATGTTGTTTTCAT |
| 18S rRNA | CATGGCTTAATCTTTGAGAC |
| U6 | GCTAATCTTCTCTGTATCGTTCCAATTTTAGTATATGTGCTGCCG |
| **Primers Used for Quantitative Real-Time PCR** | |
| **Gene** | **Primer sequence** |
| *GAPDH* |  |
| Forward | GGAGCGAGATCCCTCCAAAAT |
| Reverse | GGCTGTTGTCATACTTCTCATGG |
| *U6* |  |
| Forward | ATTGGAACGATACAGAGAAGATT |
| Reverse | GGAACGCTTCACGAATTTG |
| *circNF1* |  |
| Divergent Forward | GCAGTTTGGCCACTACAAATC |
| Divergent Reverse | AGACATTCCTTGTTGTGCTCAG |
| *circNF1* |  |
| Convergent Forward | GCTTCCAATAAAAACAGGAC |
| Convergent Reverse | GTAGTGGCCAAACTGCTGCT |
| *NF1* |  |
| Forward | TGGGACATTCGCCTCTTAAC |
| Reverse | GCAGCACTTTCTGTCAGCTGCC |
| *LinearNF1* |  |
| Forward | ACACATGCAAAATGGGAACA |
| Reverse | TGGGACATTCGCCTCTTAAC |
| *IGF2BP1* |  |
| Forward | CAACCTCAACGAGAGCGTGA |
| Reverse | CAGGCTGTCCAGTACTTCCC |
| *HMGA2* |  |
| Forward | ACCCAGGGGAAGACCCAAA |
| Reverse | CCTCTTGGCCGTTTTTCTCCA |
| *E-cadherin* |  |
| Forward | CGAGAGCTACACGTTCACGG |
| Reverse | GGGTGTCGAGGGAAAAATAGG |
| *N-cadherin* |  |
| Forward | AGCCAACCTTAACTGAGGAGT |
| Reverse | GGCAAGTTGATTGGAGGGATG |
| *Vimentin* |  |
| Forward | AGTCCACTGAGTACCGGAGAC |
| Reverse | CATTTCACGCATCTGGCGTTC |
| *ZNF460* |  |
| Forward | GGCCTTTACCTACCGCTCC |
| Reverse | GGGCTGTACTCTCATAAAAGCCT |
