# Supplementary material for: CircNF1 promotes gastric cancer metastasis by stabilizing HMGA2 mRNA through IGF2BP1 interaction
Source: Front Immunol. 2026 Feb 17;17:1767319. doi: 10.3389/fimmu.2026.1767319 (PMC12953387; doi:10.3389/fimmu.2026.1767319)
Supplement: Supplementary file 3 [file DataSheet3.docx]

**Supplementary Table 2**

**Table S2. Antibodies used in this study**

| **Antibodies** | **Source** | **Cat No.** | **Dilution** | **Molecular weight(**kDa**)** |
| --- | --- | --- | --- | --- |
| Anti- Mouse GAPDH | Proteintech | 60004-1-Ig | 1:5000 | 36 |
| Anti- Rabbit HMGA2(WB) | Abcam | ab97276 | 1:1000 | 18 |
| Anti- Rabbit IGF2BP1(WB) | Abcam | ab290736 | 1:1000 | 68 |
| Anti- Rabbit IGF2BP1(IF) | Abcam | ab290736 | 1:50 | 68 |
| Anti- Rabbit E-cadherin | Proteintech | 20874-1-AP | 1:1000 | 125 |
| Anti- Rabbit N-cadherin | Proteintech | 22018-1-AP | 1:1000 | 130 |
| Anti- Rabbit Vimentin | Proteintech | 60330-1-Ig | 1:1000 | 54 |
| Secondary goat anti-rabbit antibody | Beyotime | A0208 | 1:2000 |  |
| Secondary goat anti-mouse antibody | Beyotime | A0216 | 1:2000 |  |
