# Supplementary material for: CircNF1 promotes gastric cancer metastasis by stabilizing HMGA2 mRNA through IGF2BP1 interaction
Source: Front Immunol. 2026 Feb 17;17:1767319. doi: 10.3389/fimmu.2026.1767319 (PMC12953387; doi:10.3389/fimmu.2026.1767319)
Supplement: Supplementary file 4 [file DataSheet4.docx]

**Supplementary Figure 2**


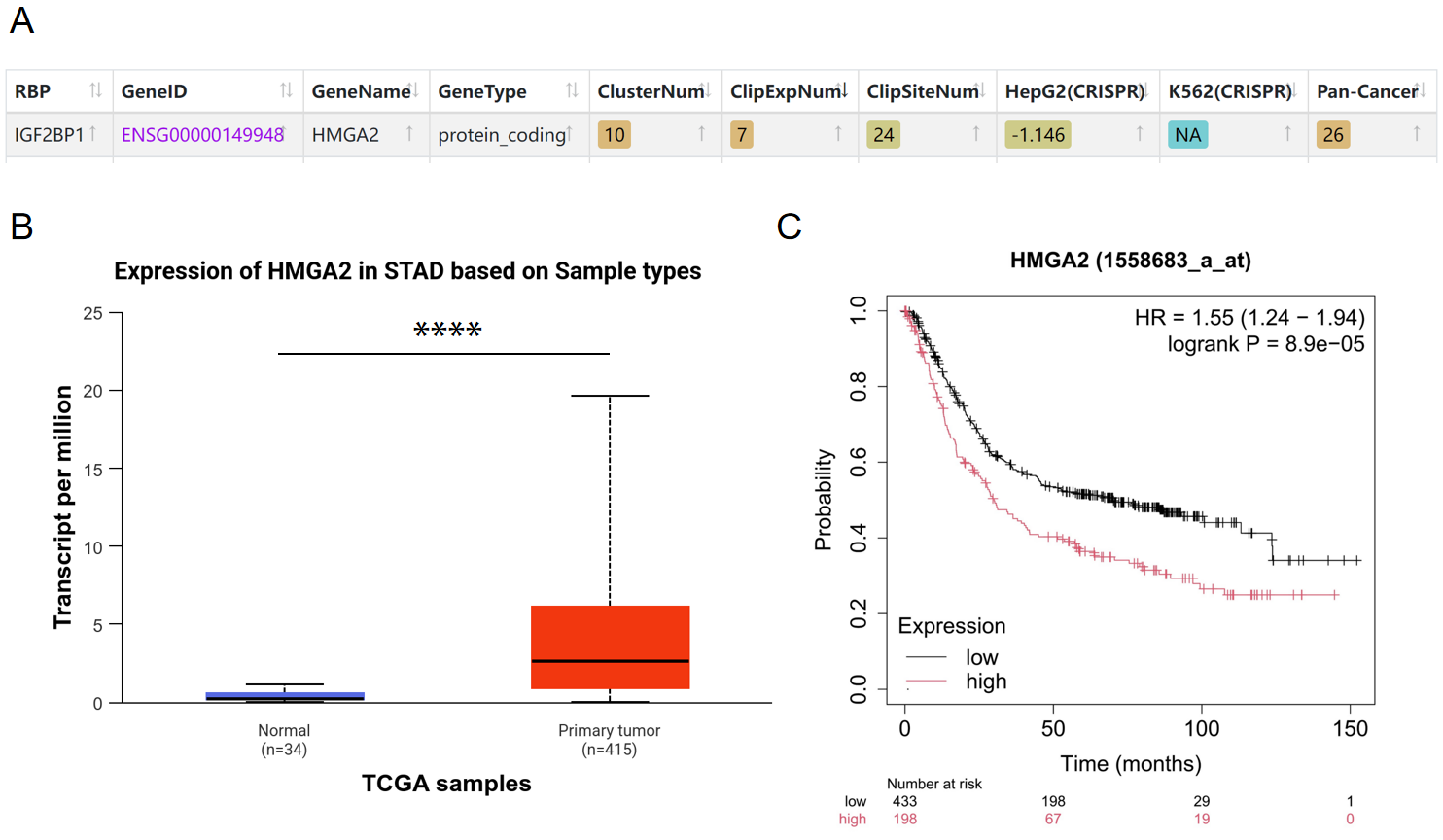


**Fig. S2 Bioinformatic analysis of IGF2BP1-HMGA2 interaction and HMGA2 clinical relevance in gastric cancer. A** Prediction of IGF2BP1-HMGA2 interaction via StarBase. **B** Box plot analysis of TCGA-STAD data showing significant HMGA2 upregulation in primary gastric tumor tissues vs. normal tissues. **C** Kaplan-Meier survival analysis demonstrating that high HMGA2 expression correlates with poor prognosis in gastric cancer patients.
